# Hypoxia-induced cancer stemness acquisition is associated with CXCR4 activation by its aberrant promoter demethylation

**DOI:** 10.1186/s12885-019-5360-7

**Published:** 2019-02-13

**Authors:** Nahyeon Kang, Su Yeon Choi, Bit Na Kim, Chang Dong Yeo, Chan Kwon Park, Young Kyoon Kim, Tae-Jung Kim, Seong-Beom Lee, Sug Hyung Lee, Jong Y. Park, Mi Sun Park, Hyeon Woo Yim, Seung Joon Kim

**Affiliations:** 1 Division of Pulmonology, Department of Internal Medicine, Seoul St. Mary’s Hospital, College of Medicine, The Catholic University of Korea, 222, Banpo-daero, Seocho-gu, Seoul, 06591 Republic of Korea; 2 The Cancer Research Institute, College of Medicine, The Catholic University of Korea, 222, Banpo-daero, Seocho-gu, Seoul, 06591 Republic of Korea; 3 Department of Hospital Pathology, College of Medicine, The Catholic University of Korea, Seoul, Republic of Korea; 4 Department of Pathology, Institute of Hansen’s Disease, College of Medicine, The Catholic University of Korea, Seoul, Republic of Korea; 5 Department of Pathology, College of Medicine, The Catholic University of Korea, Seoul, Republic of Korea; 6 Department of Cancer Epidemiology, Moffitt Cancer Center, Tampa, FL USA; 7 Department of Biostatistics, Clinical Research Coordinating Center, The Catholic University of Korea, Seoul, Republic of Korea

**Keywords:** Hypoxic stimuli, EMT, Cancer stem cell, Promoter methylation

## Abstract

**Background:**

A hypoxic microenvironment leads to an increase in the invasiveness and the metastatic potential of cancer cells within tumors via the epithelial-mesenchymal transition (EMT) and cancer stemness acquisition. However, hypoxia-induced changes in the expression and function of candidate stem cell markers and their possible molecular mechanism is still not understood.

**Methods:**

Lung cell lines were analyzed in normoxic or hypoxic conditions. For screening among the stem cell markers, a transcriptome analysis using next-generation sequencing was performed. For validation, the EMT and stem cell characteristics were analyzed. To determine whether an epigenetic mechanism was involved, the cell lines were treated with a DNA methyltransferase inhibitor (AZA), and methylation-specific PCR and bisulfite sequencing were performed.

**Results:**

Next-generation sequencing revealed that the CXCR4 expression was significantly higher after the hypoxic condition, which functionally resulted in the EMT and cancer stemness acquisition. The acquisition of the EMT and stemness properties was inhibited by treatment with CXCR4 siRNA. The CXCR4 was activated by either the hypoxic condition or treatment with AZA. The methylation-specific PCR and bisulfite sequencing displayed a decreased CXCR4 promoter methylation in the hypoxic condition.

**Conclusions:**

These results suggest that hypoxia-induced acquisition of cancer stem cell characteristics was associated with CXCR4 activation by its aberrant promoter demethylation.

## Background

Despite the improvement of cancer treatment, lung cancer has the most extensive worldwide fatality rate. In addition to surgery, chemotherapy is also an important therapeutic strategy for cancer, but it has limitation to remove all tumor cells [1, 2]. The failure of cancer treatment is associated with recurrence and metastasis. It has been reported that both EMT and cancer stemness acquisition play an important role in invasion, metastasis and chemoresistance in solid tumors [3].

Hypoxia is a major feature of solid tumors that can promote the invasion and metastatic potential of tumor cells, which results from epithelial-mesenchymal transition (EMT) and the acquisition of stemness in multiple cancers. Tumor hypoxia may result in aggressive phenotypes and poor response to therapy [4].

The EMT is a process in which cells lose their epithelial cell phenotype and convert to a mesenchymal phenotype with high motility. During this transition, epithelial cells acquire migratory and invasive properties to become cells with stem-like characteristics [5]. Hypoxia-induced EMT may be related to the acquisition of stemness by cancer cells in solid tumors. The EMT and stemness acquisition may also be related to drug resistance, which is a factor that is strongly associated with poor prognosis [6].

Cancer stem cells (CSCs) are cancer cells found in blood cancers and solid tumors, and they are important for the EMT. CSCs have the ability to self-renew and differentiate like normal stem cells. Unlike normal stem cells, these cells also have tumor-forming abilities. CSCs can be initiated from epigenetic regulation where proteins involved in the establishment and maintenance of DNA methylation have also been identified as drivers of CSC formation [7, 8]. However, the properties and mechanisms of cancer stemness acquisition have not yet been fully defined.

The aim of the present study was to evaluate the effect of hypoxia on the potential stem cell markers in the development of the EMT and cancer stemness acquisition. Moreover, their possible molecular mechanisms were also investigated.

## Methods

### Cell culture

A normal human lung cell line (BEAS-2B) and four lung cancer cell lines (A549, H292, H226, and H460) were obtained from the American Type Culture Collection (ATCC, Manassas, VA). The BEAS-2B cells were maintained in a DMEM/F12 medium; whereas the A549, H292, H226, and H460 cells were cultured in a RPMI1640 medium. The cultured medium was supplemented with 10% fetal bovine serum (FBS), 100 U/mL penicillin, 100 μg/mL streptomycin, and 250 ng/mL amphotericin B at 37 °C under 5% CO_2_ conditions. To investigate the effect of hypoxia, the cells were transferred to the Thermo Forma Anaerobic System Model 1029 (i.e., less than 1% of O_2_ partial pressure) (Thermo Electron Corp., Waltham, MA).

### Small interfering RNA (siRNA) transfection

CXCR4 siRNA (Santa Cruz Biotechnology, Santa Cruz, CA) was used to reduce the CXCR4 expression. After being cultured in a hypoxic condition for 24 h, the cells were transfected with 300 nM CXCR4 siRNA or a control siRNA using a transfection reagent (Santa Cruz Biotechnology, Santa Cruz, CA) for 6 h. The cells were harvested at 72 h post-transfection and used for the Matrigel invasion and soft agar colony formation assays.

### Transcriptome analysis with next-generation sequencing

The transcriptomes of the BEAS-2B and A549 cells that were cultured under normoxic or hypoxic conditions for 24 h were analyzed. The illumina-compatible libraries of mRNA were constructed using the TruSeq Stranded mRNA Library Preparation Kit (Illumina Inc., San Diego, CA) according to the manufacturer’s instructions. Samples were sequenced using the HiSeq 2000 Sequencing System (Illumina Inc., San Diego, CA). To estimate the expression levels, the RNA-Seq reads were mapped to the human genome using TopHat (version 1.3.3). The reference genome sequence (hg19, Genome Reference Consortium GRCh37) and annotation data were downloaded from the University of California Santa Cruz (UCSC) website (https://genome.ucsc.edu/). The transcript counts at the gene level were calculated, and the relative transcript abundances were measured in fragments per kilobase of exon per million fragments mapped (FPKM) using Cufflinks software (version 1.2.1).

### Western blot for EMT marker and CXCR4 analysis

Incubated cells were lysed in radioimmunoprecipitation assay (RIPA) buffer with protease inhibitors for 20 min. The protein samples (20 μg) were separated on a discontinuous PAGE gel and transferred to a nitrocellulose membrane at 70 V for 2 h. The membrane was then incubated with the E-cadherin, N-cadherin, fibronectin, vimentin, and α-SMA antibodies (Santa Cruz Biotechnology, Santa Cruz, CA) at 4 °C overnight. To investigate the time-dependent manner of CXCR4 expression, cells were harvested at 2, 6, 24, and 48 h after incubation. After the protein extraction, the membranes with 20 μg of protein were incubated with the CXCR4 antibodies (Santa Cruz Biotechnology, Santa Cruz, CA) at 4 °C overnight. The target protein was detected using an ECL Kit (Amersham Pharmacia Biotech, Little Chalfont, Buckinghamshire, UK) and X-ray film.

### RNA extraction and reverse transcription-polymerase chain reaction (RT-PCR) of CXCR4

The total RNA was extracted and converted into cDNA using HyperScript RT Mastermix (GeneAll Biotechnology, Seoul, Korea). The cDNA was amplified in a MyCycler Thermal Cycler (Bio-Rad, Hercules, CA) using CXCR4 forward primer: TTCTACCCCAATGACTTGTG and reverse primer: ATGTAGTAAGGCAGCCAACA. The cycling conditions involved 35 cycles for 30 s at 94 °C, 60 °C, and 72 °C. The amplified PCR products were visualized on a 1% agarose gel stained with ethidium bromide.

### Matrigel invasion assay

The Matrigel invasion assay was performed using the Corning Matrigel Matrix (Corning Inc., Tewksbury, MA). After incubation under hypoxic conditions for 24 h, 2.5 × 10^4^ cells were plated onto the Matrigel-coated membrane in Falcon Cell Culture Inserts for 24-well plates (pore size, 8 μm; Corning Inc., Corning, NY) with a 500 μL serum-free medium. Inserts were placed in 24-well culture plates containing 750 μL medium including 5% FBS. After a 24-h incubation, cells that had migrated through the Matrigel were stained with Diff-Quik solution. The cells were captured by a Leica SCN400 Slide Scanner (Leica Microsystems, Buffalo Grove, IL) and the number of invaded cells was counted in five random fields using ImageJ software. The assay was performed in triplicate.

### Soft agar colony formation assay

Lung cell lines (1 × 10^5^ cells per well) were seeded in six-well plates and incubated in a hypoxic chamber for 24 h. After cells were detached with trypsin-EDTA, 1 × 10^3^ cells per well were mixed with 0.7% agarose in 10% FBS and plated on top of 0.8% agarose in a six-well plate. After 3 weeks, colonies were washed with a phosphate-buffered saline (PBS) and stained for 2 h with 0.005% crystal violet. The images were captured using a digital camera and inverted light microscopy.

### Sphere formation assay

The lung cell lines were detached to form a single cell suspension and were seeded at densities of 5 × 10^3^ cells per well in serum-free DMEM/F12 medium supplemented with 1 x B27 (Gibco, Grand Island, NY), 20 ng/mL epidermal growth factor (EGF; PeproTech Inc., Rocky Hill, NJ), and 20 ng/mL fibroblast growth factor (FGF; PeproTech Inc., Rocky Hill, NJ) into Corning Ultra-Low Attachment Products (six-well; Corning Inc., Corning, NY). Experimental groups were incubated under hypoxic conditions for 24 h; whereas, the control groups were maintained under normoxic conditions. All cell lines were then incubated under normoxic conditions for 5 days.

### In vivo mouse tumor model

Animal experiments were performed according to guidelines approved by the Institutional Animal Care and Use Committee of the Catholic University Medical School (Approval no. CUMC-2015-0080-01). We used a xenograft tumor model for stemness investigation based on methods described in previous studies which reported the stemness characteristics using cancer cells from animal subcutaneous implantation models [9–11]. Male BALB/c nude mice (Charles River Laboratories, Yokohama, Japan) were used. After 24 h of incubation of the BEAS-2B, A549, and H226 cells in a normoxic or hypoxic chamber, 1 × 10^5^, 1 × 10^6^, or 5 × 10^6^ cells were injected subcutaneously into each side of the mouse flank: the cells incubated under normoxic conditions were implanted on the left, and the cells incubated under hypoxic conditions were implanted on the right. Tumor development and growth were monitored three times per week. Tumor sizes were measured and calculated according to the formula (L x W x H)/2, where L is the maximal length of the tumor, W is the maximal width perpendicular to L, and H is the maximal height [12]. The mice were sacrificed by CO_2_ asphyxiation to obtain tumor tissues after 2 weeks of injections.

### Immunohistochemistry of CXCR4

For CXCR4 immunohistochemistry, the paraffin sections were immunostained with an anti-CXCR4 antibody (3 μg/ml dilutions; Abcam, Cambridge, UK; #ab58176) [13]. The tissue slides were stained with H&E for comparison with the immunostained tissues, and they were reviewed by three pathologists (TJ Kim, SB Lee, and SH Lee). A semi-quantitative scoring system was used that was based on the summation of scores for staining intensity (0, negative; 1, weak; 2, moderate; 3, strong) and the percentage of positive cells (0, < 10% of tumor cells; 1, 10–25% of tumor cells; 2, 26–50% of tumor cells; 3, > 51% tumor cells).

### Immunofluorescence of EMT markers and CXCR4

Immunofluorescence was performed to analyze the representative expression patterns of the epithelial cell phenotype (E-cadherin), the mesenchymal cell phenotype (α-SMA), and a stem cell marker (CXCR4). These cells were triple-stained with E-cadherin, α-SMA, and CXCR4. The stained slides were visualized with a LSM 510 META Laser Scanning Microscope (Carl Zeiss, Jena, Germany) and analyzed with the ZEN 2009 Light Edition software (Carl Zeiss, Jena, Germany).

### Treatment with a DNA methyltransferase inhibitor

To determine whether DNA methylation influences gene expression, cells were incubated with 5 μM 5-azacytidine (AZA; DNA methyltransferase inhibitor; Sigma, St. Louis, MO) for 24 h [14].

### Real-time RT-PCR to analyze CXCR4 expression

One microgram of the RNA extracted from cell lines was used to synthesize cDNA using HyperScript™ RT Master mix (GeneAll Biotechnology, Seoul, Korea). The expression of CXCR4 was then quantified using GoTaq® qPCR Master Mix (Promega, Madison, WI) and above CXCR4 primers on Exicycler™ 96 (Bioneer, Daejeon, Korea) with an initial denaturation step of 95 °C for 5 min, followed by 40 cycles for 30 s at 95 °C, 60 °C, and 72 °C. The expression of CXCR4 was normalized to that of GAPDH.

### Methylation-specific PCR and bisulfite sequencing of the CXCR4 promoter region

The methylation-specific PCR was performed to analyze CXCR4 CpG sites in bisulfite-modified DNA [14]. The following primer pairs were used: forward, GGACGTAGTTTTTCGGTTTC; and reverse, AACGCGCAAACAATACTTT. The methylation level was normalized with the β-actin gene. A region of β-actin devoid of any CpG dinucleotide was amplified using the following primers: forward, TGGTGATGGAGGAGGTTTAGTAAGT; and reverse, AACCAATAAAACCTACTCCTCCCTTAA. The bisulfite sequencing was preformed using the following primers: forward, AAGATTGGTAGGTGTAAGTG; reverse, TTTCATTTCCTCACTCTCCC.

### Statistical analysis

Summary statistics are presented as number with percentages or ratios of categorical variables and as means with standard error of continuous variables. Categorical variables were compared with the Fisher’s exact test and one-sided Wilcoxon rank sum test for continuous variables. All statistical analyses were performed by one-tailed test because only one direction of hypothesis was of interest. An adjustment for multiple tests was not applied in our analysis. All statistical analyses were performed using SAS version 9.4 (SAS Institute, Cary, NC, USA). *P* values of less than 0.05 or less than 0.01 were considered statistically significant.

## Results

### Transcriptome analysis of EMT and stem cell markers

**Fig. 1:**
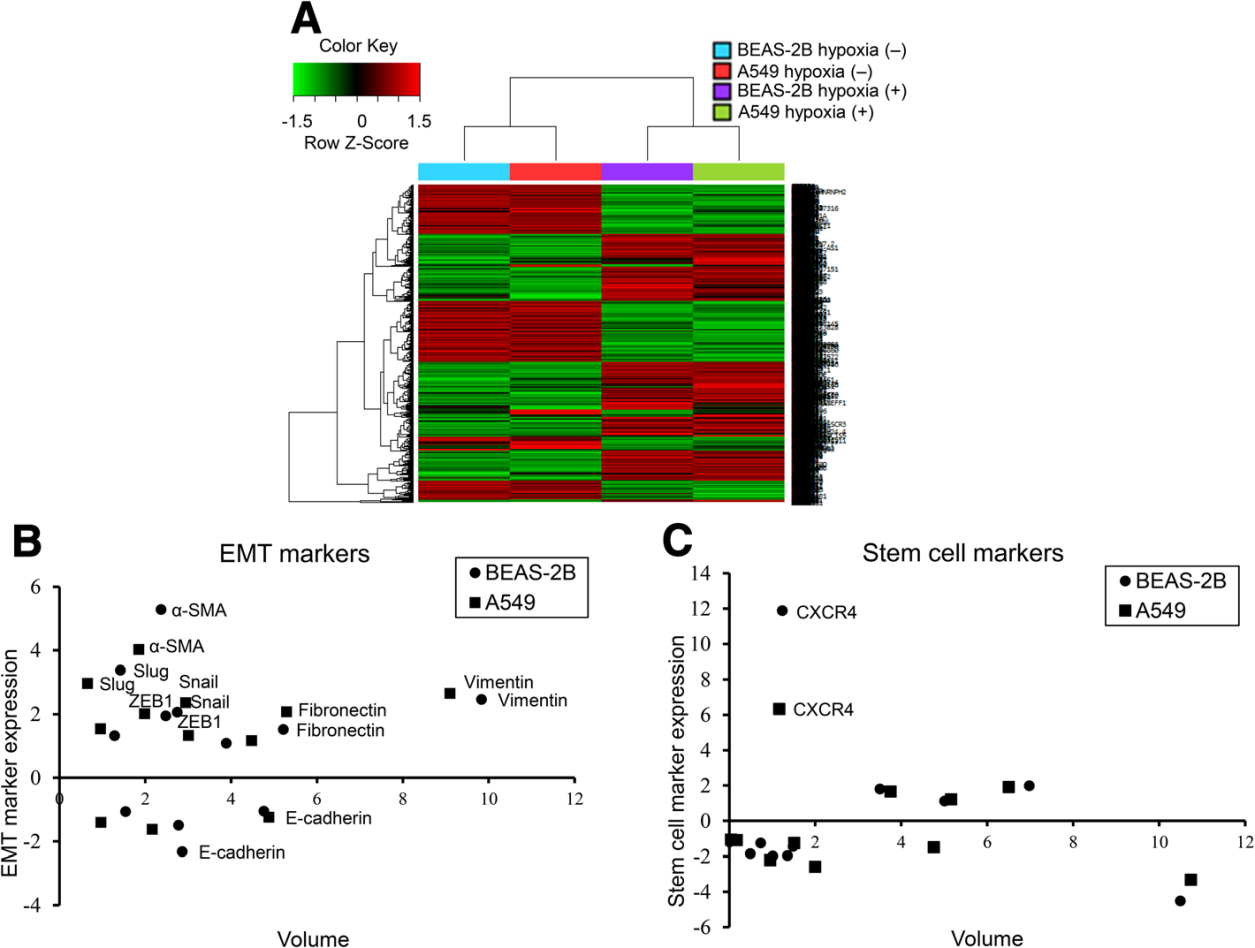
Transcriptome analysis of the BEAS-2B and A549 cells following hypoxic stimuli for 24 h using next-generation sequencing. **a** Heat map of the hierarchical clustering shows a distinct separation of mRNA expression patterns of the cells cultured under hypoxic and normoxic conditions. **b** Levels of mRNA encoding fibronectin, vimentin, α-SMA, Slug, Snail, and ZEB1 were highly induced in cells cultured in hypoxic compared with normoxic conditions; whereas, E-cadherin decreased when the cells were exposed to hypoxic stimuli. **c** Among the stem cell markers, the expression of CXCR4 increased following hypoxic stimuli in both the BEAS-2B and A549 cells

To examine the effect of hypoxia on the mRNA expression in the BEAS-2B and A549 cells, a transcriptome analysis was performed using next-generation sequencing. Distinct differences in mRNA expression patterns were observed between the cells that were cultured under normoxic and hypoxic conditions (Fig. 1a). To examine the effect of hypoxia on the EMT, various EMT markers were analyzed. Mesenchymal markers (fibronectin, vimentin, α-SMA, slug, snail, and ZEB1) increased more than 2-fold; whereas, the expression of the epithelial marker E-cadherin was reduced 1.2- to 2.3-fold in cells exposed to the hypoxic conditions (Fig. 1b). Among the cancer stem cell candidates, the fold change in the CXCR4 expression was the highest following hypoxia treatment (BEAS-2B 11.88424 and A549 6.338601) (Fig. 1c). The fold changes of the various EMT and stem cell markers are provided in Table 1.

**Table 1:** Fold changes of EMT and stem cell markers induced by hypoxia using next-generation sequencing

	Fold change		Gene volume	
Gene	BEAS-2B	A549	BEAS-2B	A549
EMT related				
**E-cadherin**	**−2.321846**	**−1.24658**	2.862953	4.882581
N-cadherin	1.082626	1.331658	3.891183	3.008228
**Fibronectin**	**1.51678**	**2.074191**	5.21957	5.292675
**Vimentin**	**2.461523**	**2.649509**	9.833378	9.097426
**α-SMA**	**5.27888**	**4.027409**	2.37067	1.848955
**Slug**	**3.376403**	**2.962488**	1.422036	0.659522
**Snail**	**2.064503**	**2.359432**	2.745241	2.941692
Twist1	−1.065424	−1.4102	1.543533	0.969468
Twist2	− 1.493418	− 1.6265	2.778423	2.162327
**ZEB1**	**1.949302**	**2.012616**	2.478841	1.987502
ZEB2	1.325055	1.536987	1.286106	0.96196
ZO-1	−1.053172	1.168809	4.765156	4.477092
Stem cell related				
CD44	1.983674	1.908933	6.979291	6.502286
**CXCR4**	**11.88424**	**6.338601**	1.237284	1.165821
ABCG2	−1.958694	−2.58677	1.357162	2.001303
ALDH1A1	−4.519745	−3.31873	10.49759	10.74185
EpCAM	−1.988084	−1.49956	1.015211	4.758595
CD90	−1.252799	−1.08908	0.732683	0.177706
Nanog	−1.023746	−1.06456	0.036569	0.044168
SOX2	−1.850566	−2.22392	0.491689	0.956587
SSEA4	−1.451824	−1.24891	1.488286	1.510724
CD166	1.117535	1.219265	5.011018	5.161295
BMI-1	1.800887	1.659949	3.508488	3.755616

EMT and stem cell markers more than 2–fold changes were marked in bold

### Expression of hypoxia-induced EMT markers and stem cell markers

**Fig. 2:**
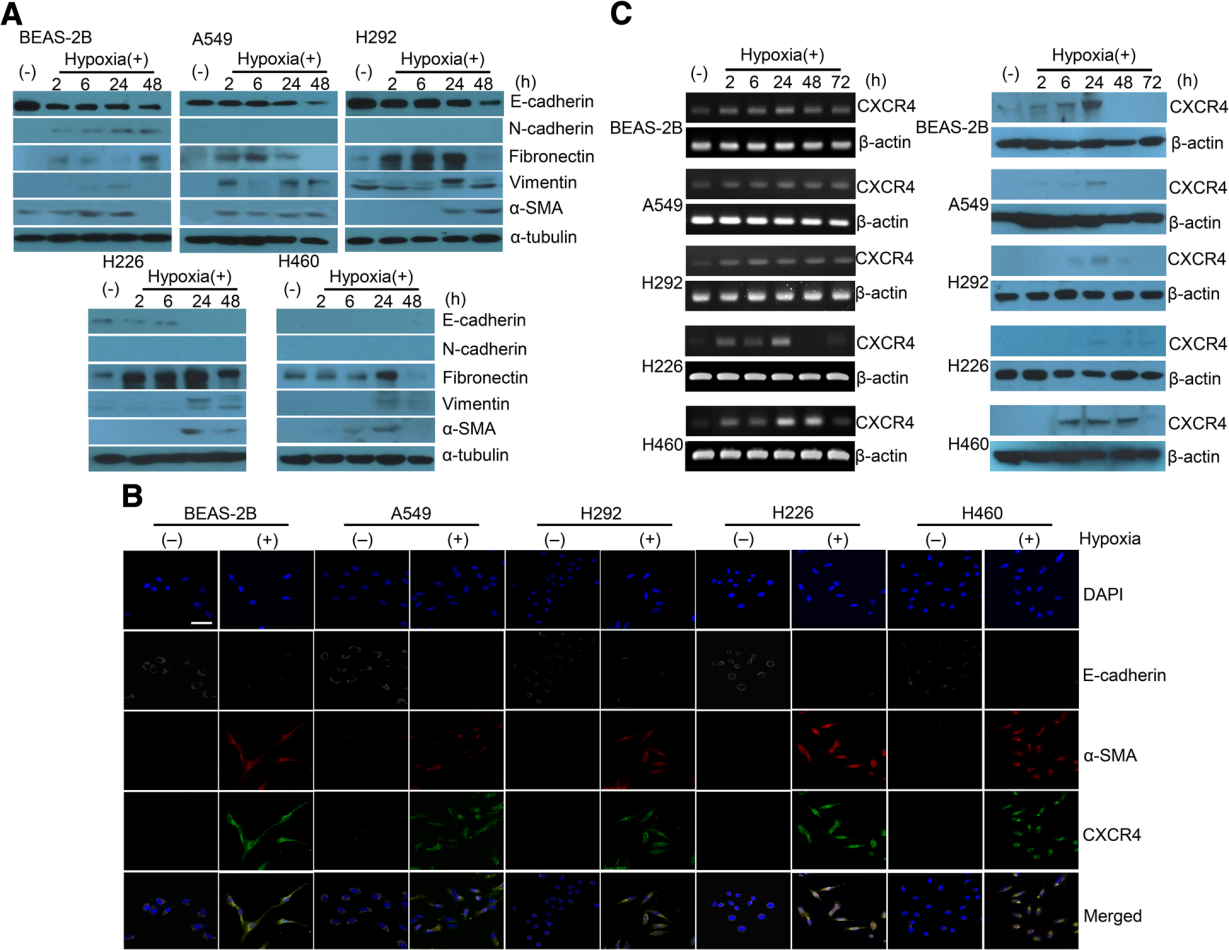
Expression of hypoxia-induced EMT markers and stem cell markers. **a** E-cadherin expression decreased according to the length of exposure to hypoxia in four lung cell lines (BEAS-2B, A549, H292, and H226). Expression of fibronectin, vimentin, and α-SMA increased; although, the expression levels differed according to the duration of exposure to hypoxic stimuli. **b** Confocal microscopy images of E-cadherin, α-SMA, and CXCR4 expression. Expression of the epithelial cell marker E-cadherin was lost following hypoxic stimuli; although, the expression of the mesenchymal cell marker α-SMA and the stem cell marker CXCR4 increased following hypoxic stimuli. E-cadherin (gray), α-SMA (red), CXCR4 (green), and DAPI (blue) (scale bar = 50 μm). **c** The time-dependent mRNA and protein expressions of CXCR4 are shown. Compared with the normoxic condition, the cells exposed to the hypoxic condition displayed increased CXCR4 mRNA and protein expressions. The mRNA expressions of CXCR4 in each cell line increased as early as 2 h; although, the protein expressions were definite in 24 or 48 h according to the cell lines

Consistent with the transcriptome analysis, the E-cadherin expression in four lung cell lines (BEAS-2B, A549, H292, and H226) decreased according to the length of time that the cells were exposed to hypoxia. The expression of fibronectin, vimentin, and α-SMA increased; although, the expression levels differed according to the length of exposure to hypoxia (Fig. 2a).

The immunofluorescence analysis revealed that the expression of the epithelial cell marker E-cadherin was lost following hypoxic stimuli; although, the expression of the mesenchymal cell marker α-SMA and stem cell marker CXCR4 was apparent following exposure to hypoxia (Fig. 2b). These results indicate that hypoxic conditions result in a gain of the EMT and stem cell phenotypes. Furthermore, cells incubated in the hypoxic chamber displayed spindle-shaped morphological changes that are consistent with the development of the EMT process.

The time-dependent mRNA and protein expressions of CXCR4 are provided in Fig. 2c. Compared with the normoxic condition, the cells exposed to the hypoxic condition exhibited increased CXCR4 mRNA and protein expressions. The mRNA expressions of CXCR4 in each of the cell lines increased as early as 2 h; although, the protein expressions were definite in 24 or 48 h according to the cell lines.

### Functional assessment of the EMT following hypoxic stimuli

**Fig. 3:**
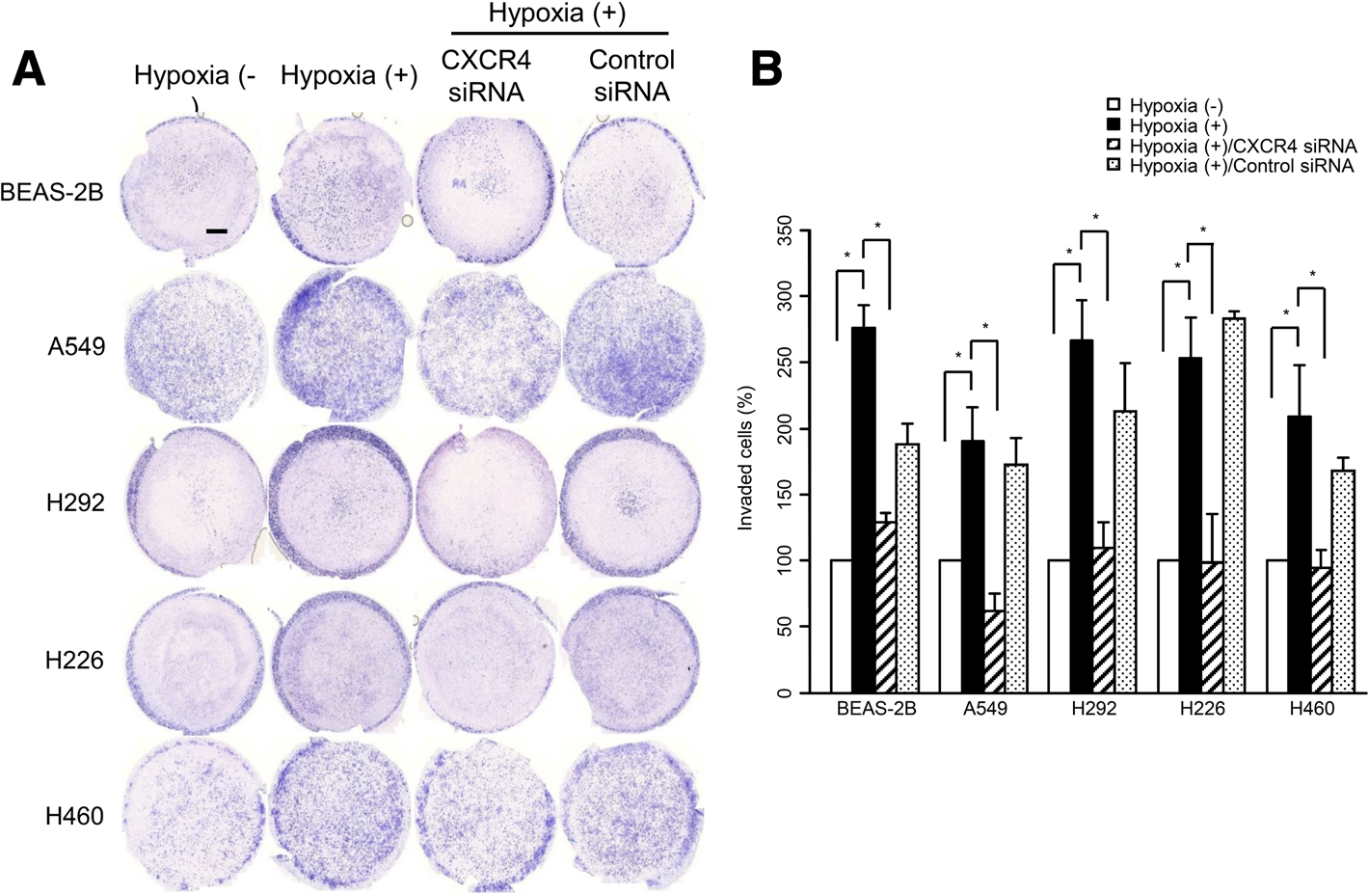
Functional assessment of the EMT with Matrigel invasion assays following hypoxic stimuli. **a**, **b** Matrigel invasion assay signifies the hypoxia enhanced transwell invasion in the BEAS-2B, A549, H292, H226, and H460 lung cells, and these increases were reduced after CXCR4 siRNA treatment. Data are presented as mean ± SE (*n* = 3, ^*^P < 0.05) (scale bar = 1000 μm)

To investigate whether hypoxia enhances invasion, Matrigel invasion assay was performed. The Matrigel invasion assay demonstrated that hypoxia significantly enhanced transwell invasion in lung cells. Moreover, compared with the hypoxia (+) group, the CXCR4 siRNA transfected cells exhibited a significant decrease in migrated cells (Fig. 3a and b).

### Acquisition of stemness in vitro

**Fig. 4:**
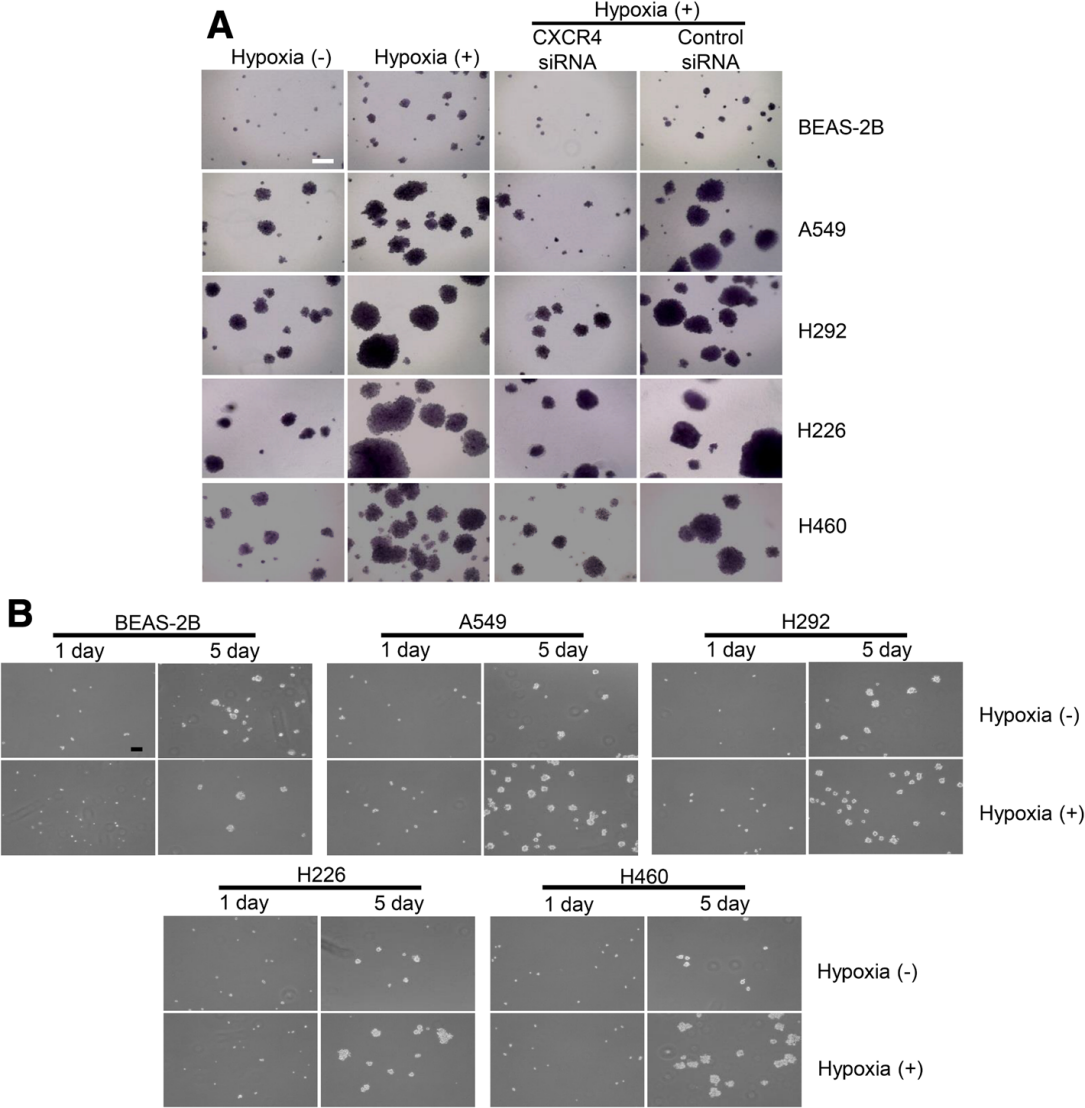
Acquisition of stemness in vitro using soft agar colony formation and sphere formation assays. **a** Colony formation rates of all lung cell lines that were incubated under hypoxic conditions were higher than those of cell lines cultured under normoxic conditions. The increased colony formation under hypoxic conditions was reduced after CXCR4 siRNA treatment (scale bar = 500 μm). **b** Lung cell lines exposed to hypoxic stimuli exhibited increased sphere formation ability compared with that of the normoxic control (scale bar = 500 μm)

The soft agar colony formation assay has been widely used to assess stem cell growth. To compare colony formation ability, cells were seeded at 1 × 10^3^ cells per well. The colony formation rates of all lung cell lines incubated under hypoxic conditions were higher than those incubated under normoxic conditions. The cells treated with CXCR4 siRNA exhibited decreased numbers and diameters in colony formation compared to the cells exposed only to hypoxia (Fig. 4a). Moreover, the lung cell lines exposed to hypoxia displayed increased sphere formation ability (Fig. 4b).

### Stemness acquisition and CXCR4 expression in vivo

**Fig. 5:**
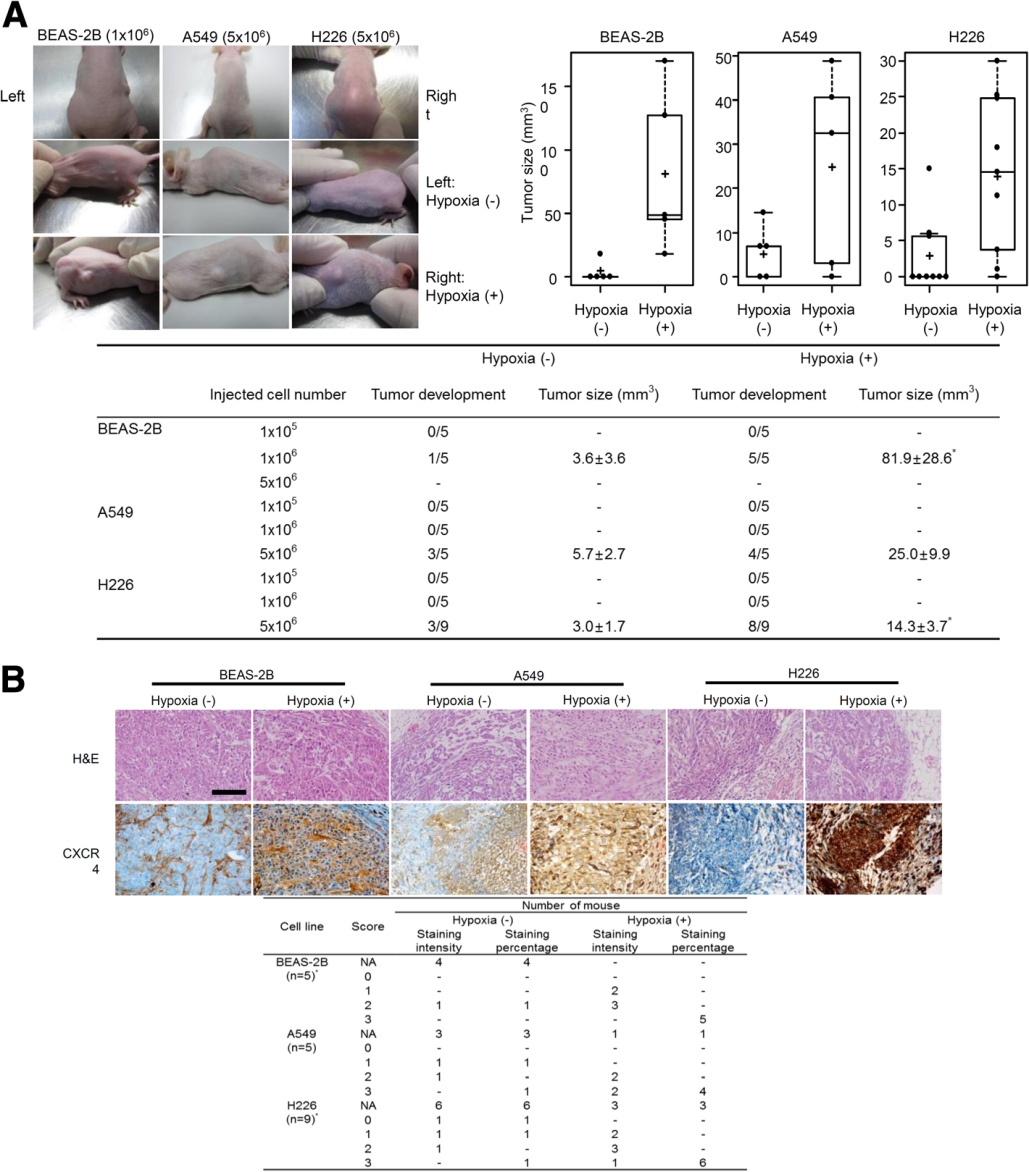
Acquisition of stemness and CXCR4 expression in vivo using a mouse tumor model. **a** Tumor-forming potential of cells cultured under the normoxic or hypoxic conditions was compared following an injection into the BALB/c nude mice. The number of mice that developed tumors was higher in the hypoxic cell group compared with the normoxic control group. In BEAS-2B or H226 cells injected mice, tumor size was significantly higher in the hypoxic cell groups compared with the normoxic groups (*P* < 0.01). A549 injected mice showed a tendency to increase although not significant. Data are presented as mean ± SE (^*^P < 0.01). **b** CXCR4 immunohistochemistry in mouse tumors injected with hypoxic cell groups showed strong CXCR4 expression compared with the normoxic controls (scale bar = 500 μm). Data are presented as mean ± SE (^*^P < 0.05)

The tumor-forming potential of the cells cultured under normoxic or hypoxic conditions was compared following an injection into BALB/c nude mice. The ability of the cells to form a tumor was evaluated as the rate of tumor development and size (Fig. 5a). In BEAS-2B or H226 cells injected mice, tumor formation and size were higher in the hypoxic cell groups compared with the normoxic groups (*P* < 0.01). A549 injected mice showed a tendency to increase although not significant.

To investigate the CXCR4 expression in mouse tumors, immunohistochemistry was performed in tumors injected with the BEAS-2B, A549, and H226 cells. Compared with the tumor tissues injected with normoxic cells, tumor tissues injected with cells exposed to hypoxia displayed strong immunoreactivity of the CXCR4 expression compared with those cultured under the normoxic conditions (Fig. 5b).

### Activation of CXCR4 by its promoter demethylation

**Fig. 6:**
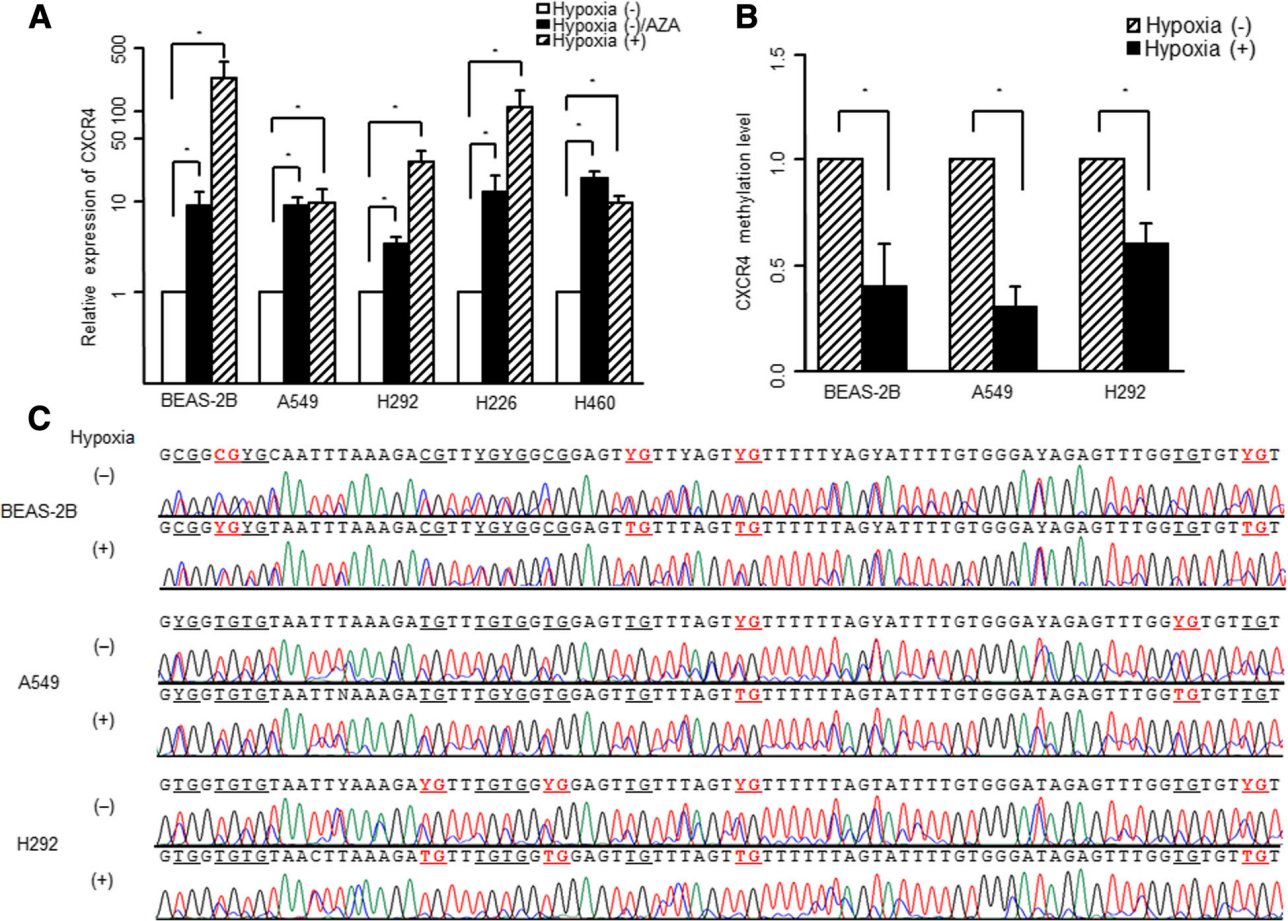
Activation of CXCR4 by its promoter DNA demethylation. **a** Real-time RT-PCR revealed significantly increased CXCR4 expression after treatment with either the DNA methyltransferase inhibitor (AZA) or hypoxic condition for 24 h compared with the controls in the lung cell lines. Data are presented as the mean ± SE (*n* = 4, ^*^P < 0.05). **b** Methylation-specific real-time PCR revealed increased promoter demethylation in hypoxic cell groups compared with normoxic controls. Data are presented as mean ± SE (*n* = 4, ^*^P < 0.05). **c** Bisulfite sequencing was performed to demonstrate the CpG site methylation status in the promoter region of CXCR4 in lung cell lines. Representative bisulfite sequencing results show 11 CpG sites presented in underlined letters within an 82-bp promoter region of CXCR4. CpG sites which were demethylated following hypoxic condition were demonstrated in red letters. Y indicates heterozygote C/T double peaks

To identify whether CXCR4 expression was activated aberrantly, we investigated the expression of CXCR4 after treatment with a DNA methyltransferase inhibitor (AZA). The AZA treated cell lines exhibited significantly increased CXCR4 expression compared with the hypoxia (−) control (*n* = 4, *P* < 0.05; Fig. 6a). These results suggest that DNA demethylation was involved in the activation of CXCR4 expression.

The DNA from the BEAS-2B, A549, and H292 cell lines was converted by sodium bisulfite, and the methylation level at the CXCR4 promoter region was compared in normoxic and hypoxic conditions using a methylation-specific PCR. The methylation-specific real-time PCR revealed a lower methylation level at the CXCR4 promoter region in the hypoxic condition compared with the normoxic condition (*n* = 4, *P* < 0.05; Fig. 6b).

Bisulfite sequencing was performed using the BEAS-2B, A549, and H292 cell lines to demonstrate the CpG site methylation status of the CXCR4 promoter region. Representative bisulfite sequencing results from 11 CpG sites presented in underlined letters within an 82-bp promoter region of CXCR4 are provided. The CpG sites that were demethylated after hypoxic conditions were demonstrated in red letters (Fig. 6c). The heterozygote C/T double peaks (Y) were observed at multiple sites.

## Discussion

The aim of the present study was to investigate the effect of hypoxia on the potential stem cell markers in the development of the EMT and cancer stemness acquisition in different lung cell lines. Furthermore, we analyzed whether the mechanism of stemness acquisition is associated with the activation of potential stem cell markers by aberrant promoter demethylation. We used various lung cell lines which have different aggressiveness to confirm the effect of hypoxia on stemness acquisition is a general feature regardless of aggressiveness of lung cancer cell lines including normal lung cell line. BEAS-2B is considered as normal lung cells. However, because BEAS-2B cells are immortalized, we investigated the effect of hypoxia on normal cells as well as cancer cells. Similar to other lung cancer cell lines, BEAS-2B also showed EMT and stemness acquisition after hypoxic stimuli. These results are consistent with previous reports, which showed induction of the EMT and stemness acquisition in BEAS-2B cell line [15–18].

In the present study, hypoxia-induced EMT was confirmed by a decrease in the epithelial marker, E-cadherin, and an increase in the mesenchymal markers, N-cadherin, fibronectin, vimentin, and α-SMA. All lung cell lines (i.e., the normal cell line BEAS-2B and four cancerous cell lines A549, H292, H226, and H460) displayed morphological changes into fibroblast-like cells, as indicated by the immunofluorescence staining. These morphological changes were functionally validated with the Matrigel invasion assay. Hypoxia enhanced the transwell invasion of the BEAS-2B, A549, H292, H226, and H460 cells. These findings are consistent with previous studies that demonstrated that hypoxia can induce the EMT [4, 19]. In addition, the CXCR4 siRNA treatment resulted in a reduction of the invasion by hypoxia.

Transcriptome analysis was performed using next-generation sequencing to investigate the effect of hypoxic stress on the expression of EMT and stem cell-related markers. Among the stem cell markers, only CXCR4 was significantly activated, and this outcome was confirmed by the RT-PCR analysis, immunofluorescence staining, and western blotting. Experimental conditions, such as oxygen concentration, duration of hypoxia exposure, and the type of cancer cell line may have influenced the experimental results.

Based on previous studies, we chose putative stem cell markers: CD44, CXCR4, ABCG2, ALDH1A1, EpCAM, CD90, Nanog, SOX2, SSEA4, CD166 and BMI-1 (Table 1). Previous reports have indicated that the chemokine receptor CXCR4 may be a candidate stem cell marker for early embryonic neural stem cells [20].

The aberrant CXCR4 levels in the nucleus and/or cytoplasm have been described in lung, breast, ovary, gastric, and esophageal cancers [21–25]. Liu et al. reported that the aberrant overexpression of CXCR4 is associated with lymph node involvement, distant metastasis, and worse overall survival in non-small cell lung cancer [26]. In the present study, CXCR4 was induced by hypoxia, which was important for the EMT and acquisition of stemness in lung cancer. The activation of CXCR4 under conditions of hypoxic stress is consistent with the findings of previous studies that hypoxic stimuli induces CXCR4 in osteosarcoma, synoviocyte and gastric cancer [27–29]. Given that, following hypoxic stimuli, CXCR4 expression was activated in all of the lung cell lines tested in the present study; thus, CXCR4 may be a major stem cell marker associated with hypoxia.

Previous studies indicated that cell proliferation and migration could be inhibited and that cell death could occur when cells are exposed to a prolonged period of severe hypoxia [30, 31]. However, cell proliferation and migration could be increased when cells were exposed to mild or moderate hypoxic stress [19, 32]. In our results, CXCR4 protein expressions increased in each cell lines exposed to hypoxia for 24 h. Based on these findings, we conducted methylation analysis in cells exposed to hypoxia for 24 h to evaluate whether DNA methylation play a role in phenotype. Evidences indicate that DNA methylation of the promoter region of CXCR4 is the primary epigenetic mechanism that regulates CXCR4 expression [33–36], and the methylation status has been proposed as a biomarker of poor patient prognosis [37, 38].

To date, little is known about the molecular mechanism of hypoxia-induced EMT and stem cell markers, and their functional association with the acquisition of stemness in lung cancer. Simonsson and Gurdon suggested that the increased expression of stem cell marker gene OCT4 coincides with its promoter demethylation, which is necessary for stemness acquisition from the somatic cell nuclei by epigenetic reprogramming [39]. The present study demonstrates that CXCR4, as a stem cell marker, was activated by hypoxic stimuli which was associated with its aberrant promoter demethylation.

## Conclusions

The results of this study suggest that hypoxia-induced EMT and cancer stemness acquisition is associated with CXCR4 activation by its aberrant promoter demethylation. However, further investigation is needed on the precise mechanism of the epigenetic regulation as well as the development of future therapeutic strategies.

## Abbreviations

AZA: Azacytidine
CSCs: Cancer stem cells
EMT: Epithelial-mesenchymal transition
